# An Unusual Case of Candida albicans Empyema in Patient With Gastric Cancer and Colon Metastasis

**DOI:** 10.7759/cureus.34023

**Published:** 2023-01-20

**Authors:** Bhavi Trivedi, Ilma Vahora, Jesus Guzman, Rohan Desai, Juan Cartagena, Aymara Y Chang

**Affiliations:** 1 Internal Medicine, Texas Tech University Health Sciences Center El Paso, El Paso, USA

**Keywords:** immunocompromised hosts, pleural effusion, fluconazole, candida albicans empyema, candida empyema

## Abstract

Candida empyema is a rare presentation seen in immunocompromised patients, with a high mortality rate if not treated appropriately. We present a rare case of *Candida albicans* empyema in a patient with gastric cancer undergoing chemotherapy who recently underwent cholecystectomy, successfully treated with fluconazole and drainage. This case not only highlights an unusual presentation of a common pathogen but stresses the fact that when patients with malignancy, present with pleural effusion, candida should be in the differential. Early detection is key in such cases, as outcomes are poor if diagnosis and treatment are delayed.

## Introduction

Candida infections are commonly reported in immunocompromised individuals and are associated with poor outcomes if not treated appropriately [1]. However, empyema caused by candida is uncommon and has a high mortality rate [2]. The common risk factors of Candida empyema include male gender, esophageal rupture, malignancy, lung or heart transplant, intra-abdominal procedures, and candidemia [3]. Given the rarity of Candida empyema and the commonality of malignant pleural effusion, this rare entity is often overlooked in patients with malignancy. Additionally, no clear guidelines exist for the management of Candida empyema [2]. We present a case of *Candida albicans* empyema in a patient with gastric cancer who recently underwent cholecystectomy, successfully treated with fluconazole and drainage.

## Case presentation

A 71-year-old female with a history of invasive poorly differentiated signet ring gastric adenocarcinoma diagnosed in 2019 and recent cholecystectomy secondary to symptomatic cholelithiasis one month prior to a presentation, presented with acute onset of oral intolerance for two days. Just prior to presentation, the patient had received palliative chemotherapy with 5-fluorouracil and leucovorin and had previously completed 10 cycles of FOLFOX (folinic acid, fluorouracil, and oxaliplatin) as therapeutic chemotherapy. Physical examination of the abdomen was significant for generalized mild tenderness on superficial palpation but no guarding or rigidity and normal bowel sounds. Vitals and labs were unremarkable. Initial chest X-ray (CXR) on admission was unremarkable for any acute pathology.

The computed tomography (CT) abdomen with contrast obtained at admission showed new areas of metastasis in the abdominal wall and the ascending colon compared to the CT abdomen from a month ago done for a cholecystectomy workup. Based on the presentation, CT findings, and cancer history, the patient was at a high risk of bowel obstruction, and a colonoscopy was performed. Colonoscopy showed a partially obstructive mass in the ascending colon, the biopsy of which revealed poorly differentiated carcinoma with signet ring features, suggestive of colon metastasis from the patient’s gastric cancer.

Initially, surgical oncology planned to perform a partial colectomy with colostomy placement. Unfortunately, the patient became hypoxic and required 3L oxygen to maintain oxygen saturation of more than 90 and developed a new up-trending leukocytosis with white blood cell (WBC) counts as high as twice that of the normal range. As a follow-up for her new onset of hypoxia, interval CXR and CT chest were obtained which showed the development of a new left-sided pleural effusion. The patient was started on prophylactic intravenous piperacillin/tazobactam and two sets of aerobic, anaerobic, and fungal blood cultures taken prior to the initiation of antibiotics did not show any growth.

**Figure 1:**
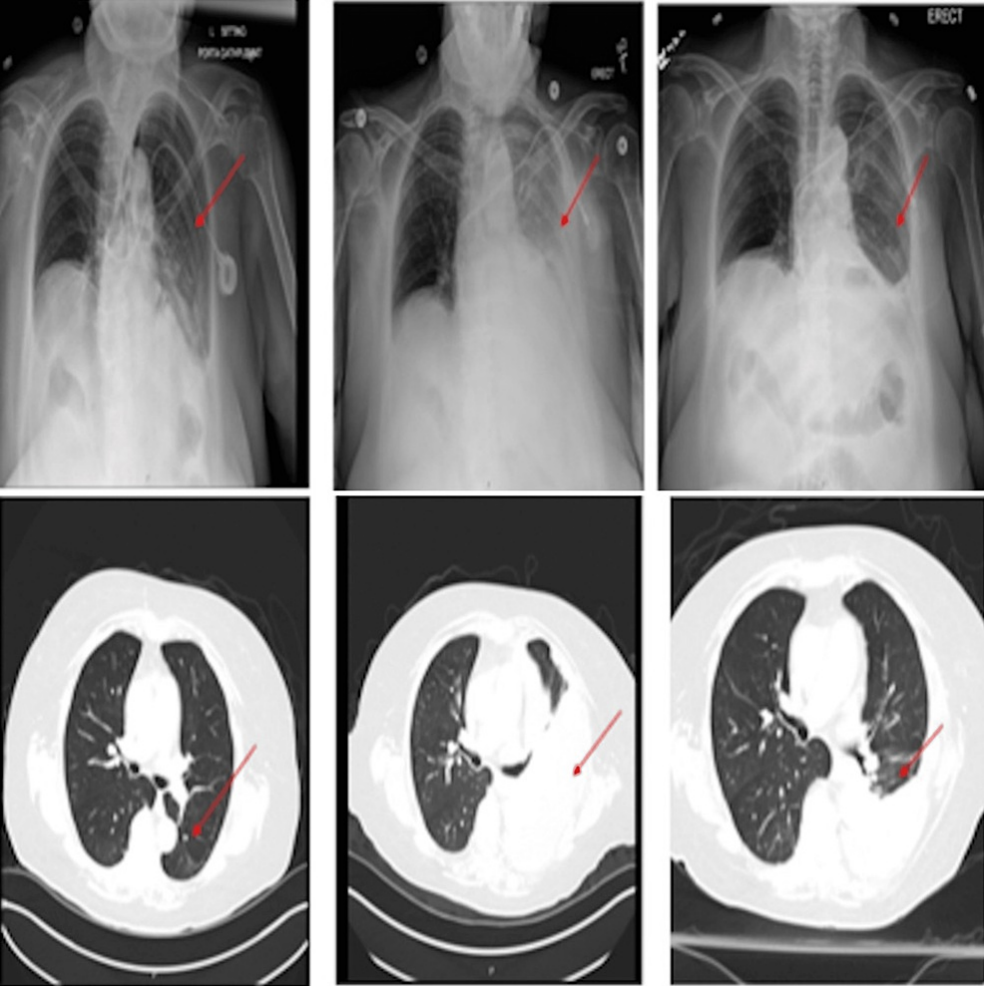
Serial chest X-ray and chest computer tomography obtained over the hospital course from left to right - Image from the day of admission (on left) showed no effusion, next image from the time patient was diagnosed with Candida empyema demonstrated the new onset of large left pleural effusion and the last images on right from post-treatment demonstrated interval resolution of empyema

A diagnostic thoracentesis was performed, and the lab analysis met light criteria for exudative effusion (Table 1). Pleural fluid cultures showed heavy growth of *Candida albicans* and the cytology report was unremarkable for malignant cells. A subsequent diagnostic and therapeutic thoracentesis with chest tube insertion was performed just two days after the first thoracentesis, prior to the initiation of antifungals. It also revealed moderate growth of *Candida albicans*. Antifungal therapy with fluconazole 400 mg daily intravenously was initiated on the day of the second thoracentesis and it was given for eight days, while the patient was admitted to the hospital. Upon initiation of fluconazole, the patient’s neutrophilic leukocytosis and tachycardia gradually resolved. Once the chest tube output was minimal, a follow-up CT chest reported interval resolution of the empyema. The chest tube was subsequently removed. Upon discharge, fluconazole 400 mg oral daily for 14 more days was prescribed to complete the course of treatment for Candida empyema. Our patient showed an excellent response to therapy with an almost complete resolution of empyema noted on a repeat CT scan and XR of the chest at end of therapy (Figure 1).

**Table 1:** Lab values LDH: lactate dehydrogenase

	Patient values	Normal rage
Serum White Count	22,000 cells/µL	4,000-11,000 cell/µL
Pleural Fluid Total Protein	3.2 g/dL	<1.5 g/dL
Exudative Pleural Fluid LDH	>8600 IU/L	> Two-thirds of upper limits of normal for serum LDH
Pleural Fluid Glucose	44 mg/dL	< 60 mg/dL
Serum Total Protein	5.2 g/dL	6.0 to 8.3 g/dL
Serum Total LDH	253 IU/L	105 to 333 IU/L

## Discussion

Candida empyema is seen typically in immunocompromised hosts and only a few cases have been reported worldwide [1]. The following criteria are required for the diagnosis of fungal empyema: (1) Isolation of a fungal species from an exudative pleural effusion; (2) Significant signs of infection, such as fever (body temperature >38.3°C) or leukocytosis (WBC >10 000 cells/µL); and (3) Isolation of the same fungal species from pleural effusion on more than one occasion [3]. In our case, *Candida albicans* was isolated from the patient's pleural effusion on two separate occasions, three days apart. The patient had significant leukocytosis with a WBC count >10,000 cells/µL, fulfilling all three criteria for diagnosis of fungal empyema.

Although uncommon, there are several risk factors for fungal empyema including thoracotomy, trauma, heart, and liver transplant, intrabdominal procedure, esophageal rupture, transdiaphragmatic spread, candidemia, and hematogenous seeding [2,4]. Malignancy and intraabdominal interventions were the risk factors that contributed to the infection in our patient. Kuo-Hsi Lin et al. found that in a 63-patient case series, malignancy was the most commonly encountered underlying disease as it was seen in 46% of patients [4]. The most common malignancies included gastrointestinal (GI) cancers such as esophageal and gastric cancer, as is seen in her patient. Infection secondary to a contiguous source was more common than a noncontiguous infection 55.6% versus 44.4% [4]. More than half the patient's had Candida empyema secondary to a GI source, with *Candida albicans* being the most common fungal pathogen isolated [4]. *Candida albicans* is one of the common opportunistic flora of the GI tract, an immunocompromised status or bridging of the mucosa, such as a cholecystectomy can predispose to candidemia and even Candida empyema through hematogenous seeding [4].

Based on previously published literature, it is evident that the patient’s immunocompromised state due to gastric cancer and chemotherapy made her prone to getting this infection. Additionally, receiving palliative chemotherapy with 5-fluorouracil and leucovorin as well as 10 cycles of FOLFOX for therapeutic chemotherapy made her even more immunocompromised and at higher risk of getting this infection. This case is unique due to concomitant Candida empyema in the setting of new-onset metastasis, which predisposed her to transient candidemia due to translocation via the GI tract due to breach of the mucosal barrier with subsequent seeding to the pleural cavity. The translocation from the gallbladder foci four weeks after her cholecystectomy is also a rare possibility.

Infectious Diseases Society of America does not provide treatment options for Candida empyema in clinical practice guidelines for the management of candidiasis (2016), likely as scarce published data is available on this topic [5,6]. There have been only four case series of fungal empyema in peer-reviewed English literature that have included 10 or more patients, only two of which focus on Candida empyema [7]. A large retrospective study of 81 patients with Candida empyema at the University of Pittsburgh Medical Center in 2021 showed that patients had better outcomes with combined treatment including pleural drainage and fluconazole compared to pleural drainage combined with any other antifungal [7]. Most patients from the study had developed Candida empyema within 90 days of undergoing thoracic or abdominal surgery, similar to our patient [7].

Common treatments for Candida empyema include fluconazole and echinocandins such as caspofungin [8]. Although caspofungin is approved by US Food and Drug Administration (FDA) for the treatment of Candida empyema, echinocandin’s effectiveness against Candida empyema and its pharmacokinetics within pleural fluid have not been studied effectively [8]. The use of echinocandins and the lack of antifungal therapy were found to be independent predictors of death in these patients [8]. Therefore, we treated the patient with drainage of the empyema along with initial parental followed by oral fluconazole till 14 days after the removal of the chest tube. Our patient showed excellent response to therapy with a successful resolution of empyema.

## Conclusions

Candida empyema is associated with high mortality in immunocompromised patients. When patients with malignancy, present with pleural effusion, candida should be in the differential, especially in the context of recent GI surgeries. Early detection is key in such cases, as outcomes are poor if diagnosis and treatment are delayed. This case report will serve as a resource for potential successful treatment with fluconazole and drainage, especially since there are no clear guidelines for treatment per the Infectious Diseases Society of America.
